# Editorial: Analysing Emotional Labor in the Service Industries: Consumer and Business Perspectives

**DOI:** 10.3389/fpsyg.2019.02290

**Published:** 2019-10-09

**Authors:** Weon Sang Yoo, Ki-Joon Back, Jungkun Park

**Affiliations:** ^1^Korea University Business School, Seoul, South Korea; ^2^Conrad N. Hilton College of Hotel and Restaurant Management, University of Houston, Houston, TX, United States; ^3^School of Business, Hanyang University, Seoul, South Korea

**Keywords:** editorial, emotion, emotional labor, emotional labor strategies, service industry (SI)

This Research Topic covers the dynamics of emotional labor and its related outcomes and antecedents in various settings. Recent consumer and service management research increasingly focuses on the role of emotions and emotional labor in service delivery and, at the same time, employees' emotional status. Frontline employees in various settings are expected to display certain emotions and suppress others in their daily interactions not only with customers but also with their managers or supervisors in order to comply with their job requirements and organizational expectations. As a result, consumers are also coping with their level of emotional acceptance when purchasing goods or receiving services, such as comfort or discomfort. Thus, this topic can be approached from both within the business and from consumer perspectives, or beyond.

There are 10 manuscripts in this Research Topic. First, despite the growing body of research on the emotional labor of service providers, little has been known about the social consequences of emotional labor by exploring double roles of emotional laborer as a consumer. Drawing on emotional dissonance theory, Park et al., investigate the relationship between the felt emotional dissonance and prosocial behavior (e.g., donation to a charity) with four experiments suggesting higher emotional dissonance serially influences perceived lack of control, emotional exhaustion, lowered sympathy for others' feeling, and subsequently lower willingness to help others.

Three manuscripts focus on the emotional relationship between consumers and service providers. Jeong et al., investigate a conceptual model articulating the nature of customer expectations and satisfaction over services with several emotional factors. Five propositions about consumer emotional service expectations as a primary antecedent toward confirmation, perceived quality, and satisfaction are provided. As moderators, two dimensions of consumer detection of emotional labor (i.e., detecting deep acting and surface acting) are imposed on each of the relationships. Evidence demonstrates the roles of emotional service expectation in service confirmation and satisfaction. The moderating effects of consumer detection of employees' emotional strategies are limited to the relationship between emotional service expectation and confirmation in the hotel/restaurant industry. Zhang et al., examine how employee surface acting relates to their sabotage to customers through the mediating role of emotional exhaustion. Also, they explore the moderating roles of coworker exchange and leader-member exchange using the conservation of resources theory and social exchange theory as conceptual frameworks, and find that coworker exchange buffers the positive effect of surface acting on emotional exhaustion. In addition, the study stresses the effect of surface acting on employee harmful behaviors, the potential underlying mechanism, and boundary conditions to mitigate the negative consequences. Lee et al., explore the relationship between employees' expression of emotional labor and perception of customer feedbacks touching how the perception of customer feedback affects emotional exhaustion in order to understand how emotional exhaustion affects job satisfaction and turnover intentions. The result suggests that employees with better understanding of their own emotions who experience a more significant negative effect when there is a discrepancy between what they feel and how they should act.

Within the organizational level, there are six articles. Based on social identity theory, Oh et al., examines the relationship between corporate social responsibility (CSR) perception and emotional labor strategies, and the effects of the interaction between CSR perception and moral identification on emotional labor strategies indicating main effect of CSR perception on emotional labor strategies. The results find CSR perception of employees is positively related to deep acting revealing employees' views on their organization's social responsiveness and morality affect their emotional labor strategies. Also, affective organizational commitment and moral identifications mediate the relationship between CSR perception and surface acting but not between CSR perception and deep acting. Lee et al., focus bond and fit toward emotional labor within organization. The manuscript examines the influences of relationship bonds on emotional labor through person-organization fit (P-O fit) and the moderating effects of collectivism between P-O fit and emotional labor in the financial industry identifying financial, social, and structural bonds enhanced P-O fit that improves deep acting. This study not only suggests the empirical evidence identifying the process of relationship bonds influencing emotional labor but also expands the scope of study by examining moderating roles of collectivism in cultural psychology aspect.

Two manuscripts focus on knowledge-based service providers. Huang et al., investigate school teachers' emotional labor process and the consequential outcomes for their well-being examining the roles of two antecedents, namely, teachers' perceptions of display rules and self-monitoring tendencies. The results show that self-monitoring generally have stronger, though maladaptive, effects than display rule perceptions on individuals' use of emotional labor strategies and well-being. Both self-monitoring and display rule perceptions are positively related to two emotional labor strategies concluding self-monitoring may be less beneficial than previously thought. Knowledge-based service workers' display rule perceptions and deep acting may not necessarily be harmful to their well-being but reflect their role identification and commitment. Han et al., investigate the effects of level of emotional labor and its impact of emotional labor on organizational trust and organizational commitment with college administrative staffs with 3 factors (i.e., job stress, intimacy, and professionalism) as determinants of emotional labor.

Cho and Cho examine the internal state of employees' emotion how people anticipate negative emotion when faced with an uncertain outcome and try to manage their expectation. While extant research streams remain equivocal on whether managing expectation always succeeds, this research examines situations in which setting a low expectation can have an adverse emotional impact and ways to alleviate this negative emotional consequence using goal setting and false-feedback paradigm. Choi et al., explore rather negative consequences of emotional problem in workplace taking a holistic approach associating the concept of bullying with firm-level performance as well as stakeholders' responses in the market analyzing whether and how market investors react to the news of corporate harassment by top officials of publicly listed firms in Korea. The results find significantly negative stock price reactions to news of corporate bullying and harassment.

We wish to express our great appreciation to the authors of this special issue for their enthusiasm and scholarly achievements. Although the individual manuscripts reflect considerable variations in focus and perspectives, we genuinely believe that there is a tremendous cohesive message for a better understanding of the filed of emotional labor and consider applying various theories to further developing effective employee retention strategies and enhancing the well-being of all stakeholders in the service delivery process.

## Author Contributions

All authors listed have made a substantial, direct and intellectual contribution to the work, and approved it for publication.

### Conflict of Interest

The authors declare that the research was conducted in the absence of any commercial or financial relationships that could be construed as a potential conflict of interest.

